# Improved survival of children with sepsis and purpura: effects of age, gender, and era

**DOI:** 10.1186/cc6161

**Published:** 2007-10-18

**Authors:** Martine Maat, Corinne MP Buysse, Marieke Emonts, Lodewijk Spanjaard, Koen FM Joosten, Ronald de Groot, Jan A Hazelzet

**Affiliations:** 1 Department of Paediatrics, Division of Infectious Diseases and Immunology, Erasmus MC-Sophia Children's Hospital, University Medical Center, Dr. Molewaterplein 60, 3015 GJ Rotterdam, The Netherlands; 2 Department of Paediatrics, Division of Paediatric Intensive Care, Erasmus MC-Sophia Children's Hospital, University Medical Center, Dr. Molewaterplein 60, 3015 GJ Rotterdam, The Netherlands; 3 Netherlands Reference Laboratory for Bacterial Meningitis, Department of Medical Microbiology, Academic Medical Center Amsterdam, Meibergdreef 15, 1100 DD Amsterdam, The Netherlands; 4 Department of Paediatrics, University Medical Center St. Radboud, Geert Grooteplein 10, 6500 HB Nijmegen, The Netherlands

## Abstract

**Background:**

To gain insight into factors that might affect results of future case-control studies, we performed an analysis of children with sepsis and purpura admitted to the paediatric intensive care unit (PICU) of Erasmus MC-Sophia Children's Hospital (Rotterdam, The Netherlands).

**Methods:**

Between 1988 and 2006, all 287 children consecutively admitted with sepsis and purpura were included in various sepsis studies. Data regarding age, gender, ethnicity, serogroup of *Neisseria meningitidis*, severity, therapy, and survival were collected prospectively. These data were pooled into one database and analyzed retrospectively.

**Results:**

The case fatality rate (CFR) from sepsis and purpura was 15.7%. During the study period, survival improved significantly. Younger age was significantly associated with more severe disease and a higher CFR. Children under the median age of 3.0 years had an increased risk of case fatality (odds ratio 4.3, 95% confidence interval 2.1 to 9.2; *p *< 0.001). Gender was not associated with CFR. However, males did have higher Paediatric Risk of Mortality scores, fewer PICU-free days, and more presence of shock. The course of sepsis and purpura was not related to ethnic origin. A causative organism was isolated in 84.3% of cases. *N. meningitidis *was the major organism (97.5%). Although *N. meningitidis *serogroup B was observed more often in younger children, serogroups were not associated with severity or survival. During the study period, the use of inotropic agents and corticosteroids changed substantially (less dopamine and more dobutamine, norepinephrine, and corticosteroids).

**Conclusion:**

Age and gender are determinants of severity of paediatric sepsis and purpura. Survival rates have improved during the last two decades.

## Introduction

Sepsis and purpura in children is a clinically distinct disease entity caused by high concentrations of microbes and their products. Since the introduction of a vaccine against *Haemophilus influenzae *type b, more than 90% of the cases of sepsis and purpura in the Western world have been caused by *Neisseria meningitidis *[1-3]. The resulting disease entity is referred to as meningococcal sepsis.

Meningococcal sepsis in children develops when the initial host response to the infection becomes inappropriately amplified and dysregulated. Clinically, the onset is often insidious. After the development of the first petechiae, the patient rapidly deteriorates and may subsequently develop shock, disseminated intravascular coagulation (DIC), and ultimately organ failure. The severity of these symptoms requires immediate therapy [4,5]. Despite recent advances in therapy, the case fatality rate (CFR) remains high and ranges from 4% to 40% [1,6-8]. The incidence of disease is highest among young children (0 to 4 years old) and adolescents [1-3]. In The Netherlands, meningococcal sepsis occurs in 4.5 per 100,000 inhabitants (2001). Due to the sudden increase in the incidence of meningococcal disease in 2001, a national vaccination campaign against serogroup C meningococci (2002) was implemented among children from 1 to 18 years of age [9,10].

In recent years, many studies have focused on the elucidation of the pathogenesis of sepsis. However, much about the epidemiology of sepsis in children is still unknown. In this paper, we seek to describe the epidemiology of sepsis and purpura in children referred to the paediatric intensive care unit (PICU) of Erasmus MC-Sophia Children's Hospital in Rotterdam, The Netherlands. The aim of this study was to analyze the variation in severity and survival of children with respect to age, gender, ethnicity, and serogroup of *N. meningitidis*.

## Materials and methods

The study was conducted in accordance with the Declaration of Helsinki. Permission for the study was obtained from the medical ethics committee of Erasmus MC.

### Participants

All children admitted with sepsis and purpura (and/or petechiae) to the PICU of the Erasmus MC-Sophia Children's Hospital since 1988 were included. A vast majority of the children were previously included in Rotterdam-based sepsis studies [11-16]. Data regarding the remaining children with sepsis and purpura were derived from PICU admission records. Informed consent was obtained from parents or legal guardians of all children who were included in this study. Children were considered to have sepsis when they presented with tachycardia, tachypnea, and a body temperature of less than 36°C or greater than 38.5°C (rectal) [17]. Prospective data on all children were collected at various time points in the course of the disease. Both laboratory parameters and disease severity scoring systems, like Paediatric Risk of Mortality (PRISM) score and predicted death rate (PDR) based on the Rotterdam score, were selected as markers of severity of disease [18-20]. Additionally, presence of DIC and presence of shock were recorded as markers of severity [17,19,21]. The number of PICU-free days was determined on day 28 after admission using the date of admission and the date of discharge. A non-survivor had 0 PICU-free days. All laboratory parameters, obtained at baseline from an arterial blood sample, were collected within 4 hours after admission to the PICU.

Ethnicity was determined by checking patient information, and if it was not specified, first and last names were checked and ethnicity was determined by means of the combined name method [22]. Ethnicity was categorized into Dutch Caucasian, Turkish, Moroccan, Hindustani, African descent, and other. Serogrouping of *N. meningitidis *isolates was performed at the Netherlands Reference Laboratory for Bacterial Meningitis Amsterdam using immunodiffusion with polyclonal antisera [23].

### Statistical analyses

Retrospectively, severity and survival of children with sepsis and purpura with respect to age, gender, causative organism, and ethnicity were analyzed by means of SPSS 11.01 (SPSS Inc., Chicago, IL, USA) Clinical and laboratory parameters were included in the analysis only if they were determined in at least 90% of all children.

Mann-Whitney *U *test, Student *t *test, chi-square test, and Spearman correlation (r_s_) were used when appropriate. When necessary, variables were log-transformed to obtain an approximately normal distribution. For these variables, geometric mean values and their 95% confidence intervals (CIs) are depicted in the text and tables. *P *values of less than or equal to 0.05 were considered statistically significant.

## Results

**Figure 1:**
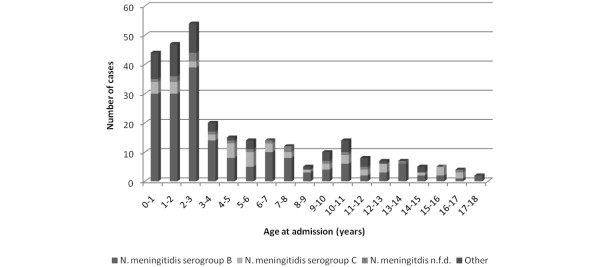
Distribution of age at admission in children with sepsis and purpura. The children are subdivided according to causative organism. *N. meningitidis*, *Neisseria meningitidis*. Not further defined (n.f.d.).

Between August 1988 and June 2006, 287 children with sepsis and purpura were admitted to the PICU of the Erasmus MC-Sophia Children's Hospital. The overall CFR was 15.7% (45 children died). The median age at admission was 3.0 years (range 0.1 to 17.9 years) (Figure 1). Of the 287 children, 155 (54%) were male and 132 (46%) were female. The male-to-female ratio was 1.2. The majority of the children were Dutch Caucasians (73.8%). Laboratory parameters present at baseline in more than 90% of the children were base excess, lactate, C-reactive protein (CRP), fibrinogen, platelet count, leukocytes, and glucose.

### Survival

**Figure 2:**
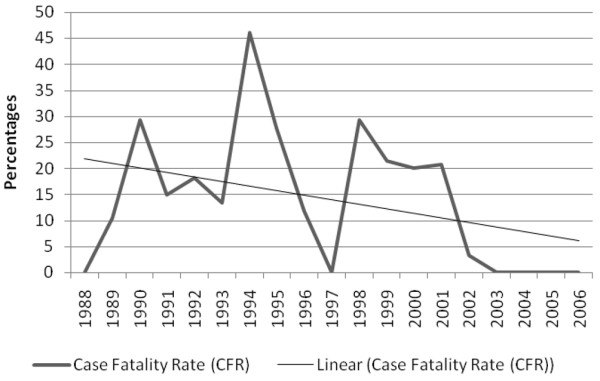
Case fatality rate (CFR) and CFR trend line during the study period.

Severity of illness was significantly less in survivors when compared with non-survivors, both in disease severity scoring systems and laboratory parameters (Table 1). Survival was significantly correlated with year of admission (*p *≤ 0.05, r_s _0.128), indicating that survival has improved significantly during the study period (Figure 2). Gender did not differ between survivors and non-survivors (*p *= 0.15). The vast majority of fatal cases died of refractory septic shock (75.6%).

**Table 1:** Comparison of disease characteristics between non-survivors and survivors

	Survivors^a^	Non-survivors^a^
Total number of children (%)	242	45

	(84.3)	(15.7)
Male-to-female ratio	1.1	1.7
Number of children with DIC (%)	174^b^	32^b^
	(75)	(97)
*Neisseria meningitidis *serogroup		
B (%)	147 (74.2)	28 (73.7)
C (%)	37 (18.7)	7 (18.4)
PRISM score	14^c^	23^c^
	(1 to 37)	(8 to 44)
Predicted death rate (%)^d^	3.1^c^	87.4^c^
	(0 to 100)	(1.1 to 100.0)
Base excess (mmol/L)	-7^c^	-13^c^
	(-23 to 4.4)	(-28 to 0.6)
Lactate (mmol/L)	3.7^c^	6.6^c^
Geometric mean, 95% CI	3.4 to 4.3	5.8 to 7.4
C-reactive protein (mg/L)	106^c^	53^c^
	(10 to 334)	(6 to 226)
Fibrinogen (g/L)	2.8^c^	0.9^c^
	(0.3 to 6.8)	(0.2 to 5.4)
Platelet count (×10^3^/μL)	126^c^	47^c^
	(15 to 475)	(13 to 202)
Leukocytes (×10^3^/μL)	10.6^c^	4.7^c^
Geometric mean, 95% CI	9.5 to 11.9	3.7 to 6.0
Glucose (mmol/L)	6.3^c^	4.3^c^
Geometric mean, 95% CI	5.9 to 6.8	3.6 to 5.3

^a^Results represent median (min-max) unless stated otherwise. ^b^*p *< 0.01.^c^*p *< 0.001. ^d^Predicted death rate was based on the Rotterdam score. CI, confidence interval; DIC, disseminated intravascular coagulation; PRISM, Paediatric Risk of Mortality.

### Age

**Figure 3:**
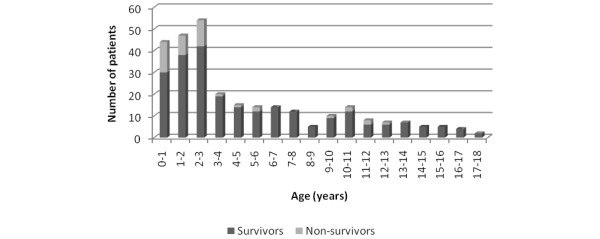
Distribution of age at admission among survivors and non-survivors of sepsis and purpura.

Age was significantly correlated with PRISM score (*p *< 0.001, r_s _-0.317), PDR (*p *< 0.001, r_s _-0.321), presence of DIC (*p *< 0.001, r_s _-0.245), base excess (*p *< 0.001, r_s _0.313), CRP (*p *< 0.05, r_s _0.161), fibrinogen (*p *< 0.001, r_s _0.301), leukocyte count (*p *< 0.001, r_s _0.284), thrombocyte count (*p *< 0.01, r_s _0.184), and glucose levels (*p *< 0.001, r_s _0.296). This indicates that younger children had higher PRISM scores, higher PDR, more presence of DIC, lower base excess, lower CRP, lower fibrinogen, lower leukocyte count, lower thrombocyte count, and lower glucose levels on admission. The median age of children was 3.0 years (range 0.1 to 17.9 years). Children 3.0 years old or younger had a higher CFR (odds ratio 4.3, 95% CI 2.1 to 9.2; *p *< 0.001) (Figure 3).

### Gender

The median age did not differ significantly between males (2.8 years) and females (3.5 years) (*p *= 0.16). Male patients had significantly fewer PICU-free days (*p *= 0.04) and higher PRISM scores (*p *= 0.02) than females. Shock was slightly more common in males than in females (89% versus 80%; *p *= 0.04). CFR and other markers of severity of disease did not differ between males and females. Because males had higher PRISM scores but no increased CFR, we analyzed the different variables determining the PRISM score. Of these variables, only a trend for lower glucose levels in males compared with females was observed (*p *= 0.06).

### Ethnicity

The majority of the children were Dutch Caucasians (*n *= 211, 73.5%). Of the remaining 76 children, 12 were Turkish (4.2%), 16 were Moroccan (5.6%), 3 were Hindustani (1.1%), 7 were of African descent (2.5%), 7 were designated other (2.5%), and in 31 children ethnicity could not be determined (10.8%). No differences with respect to severity of disease or case fatality were found between the different ethnic groups.

### Causative organism

**Figure 4:**
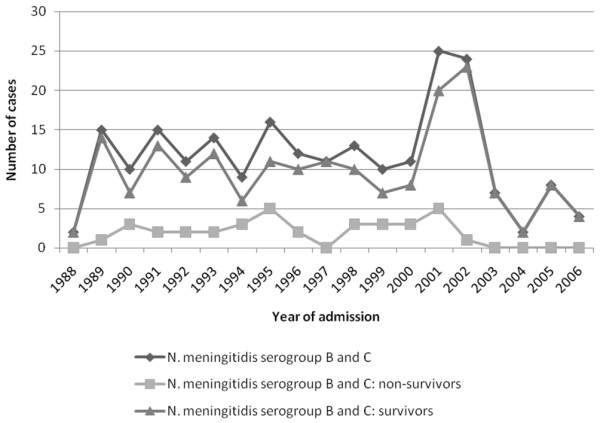
Number of children with sepsis and purpura due to *Neisseria meningitidis *per year (since 1988), admitted to the paediatric intensive care unit of Erasmus MC-Sophia Children's Hospital (Rotterdam, The Netherlands).

A causative organism could be determined in 242 children (84.3%), with *N. meningitidis *being the major causative organism (*n *= 236, 97.5%) (Figure 4). Of these 236, 175 (74.2%) were *N. meningitidis *serogroup B, 44 (18.6%) were serogroup C, and in 17 (7.2%) the serogroup was not determined (Table 2). *Streptococcus pneumoniae *was the causative organism in 3 children, *Staphylococcus aureus *in 1, and *H. influenzae *in 2. Of the remaining 45 children, 43 had clinical features of meningococcal sepsis [3].

**Table 2:** Incidence of serogroup, serotype, and serosubtype of *Neisseria meningitidis*

Serogroup	Serotype	Serosubtype	Number	Percentage
B	1	P1.4	4	1.8
		P1.16	4	1.8
		NT	3	1.4
		Other	1	0.5
	2A		3	1.4
	4	P1.4	57	26
		P1.6	3	1.4
		P1.7	3	1.4
		P1.9	4	1.8
		P1.10	4	1.8
		P1.15	5	2.3
		NT	28	12.8
		Other	13	5.9
	NT	P1.1	6	2.7
		P1.4	8	3.7
		NT	8	3.7
		Other	4	1.8
	Other		17	7.8
C	2A	P1.2	12	5.5
		P1.5	9	4.1
		P1.7	1	0.5
		NT	7	3.2
	2B	P1.1	1	0.5
		P1.2	7	3.2
	4	P1.4	3	1.4
	NT		2	0.9
	Other		2	0.9

NT, non-typable.

For logistic reasons, the causative organism could not be determined in 2 children. No differences with respect to survival, disease severity scoring systems, and presence of shock were observed between *N. meningitidis *serogroups B and C. However, the median age of children with sepsis and purpura due to serogroup B was lower than that of the serogroup C-infected children (2.8 and 6.0 years, respectively; *p *< 0.001) (Table 3). The distribution of serogroup, serotype, and serosubtype of *N. meningitidis *in the positive cultures is depicted in Table 2.

**Table 3:** Comparison of disease characteristics based on serogroup of *Neisseria meningitidis*

	*N. meningitidis*^a^	
	Serogroup B	Serogroup C
Total number of children	175	44
Age in years	2.8^b^	6.0^b^
	(0.1 to 17.9)	(0.1 to 16.5)
PRISM score	16	14
	(1 to 37)	(1 to 35)
Predicted death rate (%)^c^	8.9	4.9
	(0 to 100)	(0 to 100)
Number of children with DIC (%)	128	32
	(81)	(74)
Number of PICU-free days	24	25
	(0 to 28)	(0 to 27)
Base excess (mmol/L)	-8	-8.0
	(-21 to 4.4)	(-28 to 3)
Lactate (mmol/L)	4.2	3.5
Geometric mean, 95% CI	3.8 to 4.6	2.9 to 4.2
C-reactive protein (mg/L)	82^d^	128^d^
	(6 to 287)	(20 to 326)
Fibrinogen (g/L)	2.4	2.8
	(0.2 to 6.8)	(0.3 to 6.6)
Platelet count (×10^3^/μL)	110	113
	(15 to 475)	(13 to 336)
Leukocytes (×10^3^/μL)	8.8^e^	12.2^e^
Geometric mean, 95% CI	7.6 to 10.1	9.9 to 15.0
Glucose (mmol/L)	5.9	6.2
Geometric mean, 95% CI	5.4 to 6.5	5.5 to 6.9

^a^Results represent median (min-max) unless stated otherwise. ^b^*p *< 0.001. ^c^Predicted death rate was based on the Rotterdam score. ^d^*p *< 0.01. ^e^*p *< 0.05. CI, confidence interval; DIC, disseminated intravascular coagulation; PICU, paediatric intensive care unit; PRISM, Paediatric Risk of Mortality.

### Meningococcal C vaccination campaign and therapy

In 2001 and 2002, a sudden increase was noted in the incidence of meningococcal infection in The Netherlands. This was caused mainly by serogroup C *N. meningitidis*. The implementation of the meningococcal C vaccination campaign in July 2002 resulted in a sharp decline in the number of cases caused by serogroup C (Figure 4). Since 2003, there has not been a case of sepsis and purpura due to *N. meningitidis *serogroup C in our hospital. Parallel to this, the incidence of serogroup B has declined and is returning to the incidence level of before 1989. Before the national meningococcal C vaccination, 248 children in our study population were admitted with sepsis and purpura; since the vaccination campaign, 39 children have been admitted.

**Figure 5:**
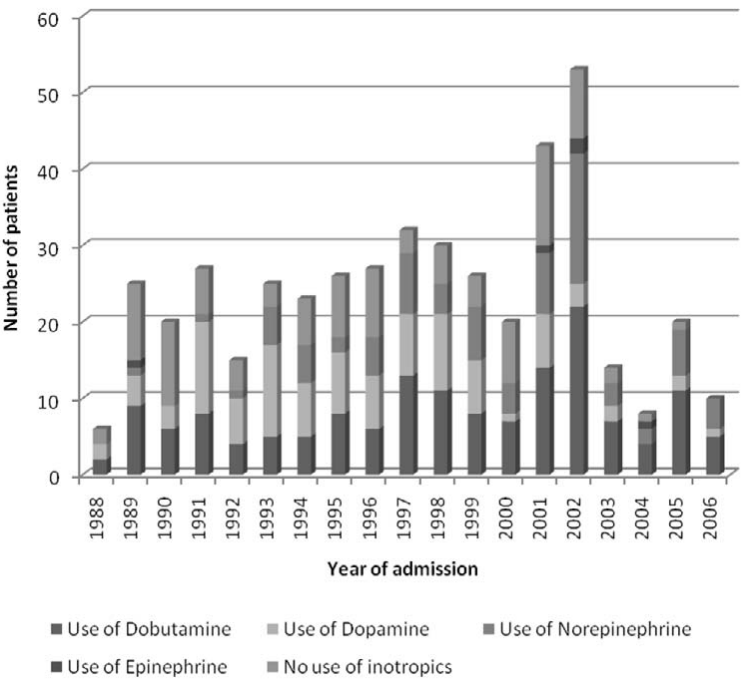
Use of inotropic agents during the study period 1988 to 2006. Some patients received more than one inotropic agent. Therefore, the number of patients in this figure exceeds the number of patients in this study (*N *= 287).

Remarkably, since the implementation of meningococcal C vaccination, no deaths have occurred in children with sepsis and purpura admitted to our PICU. The median age of the children did not differ significantly before and after vaccination (3.2 and 2.5 years, respectively; *p *= 0.23) (Table 4). Glucose levels were significantly lower in the patient group before the vaccination campaign compared with the patient group after (*p *< 0.05). Children admitted before the vaccination campaign had significantly fewer PICU-free days and more presence of DIC (both *p *< 0.05). The PRISM score was not significantly different between patient groups before and after the meningococcal C vaccination campaign. In addition, since 2002, treatment of children with meningococcal sepsis at our PICU has changed due to the implementation of international guidelines [8]. After the vaccination campaign, more children were treated with corticosteroids (18 [9.3%] before versus 15 [42.9%] after; *p *< 0.001) and more children were mechanically ventilated (128 [51.8%] before versus 28 [71.8%] after; *p *< 0.05) (Table 3). In addition, year of admission was significantly correlated with the use of dobutamine (*p *< 0.001, r_s _0.262), dopamine (*p *< 0.001, r_s _-0.218), norepinephrine (*p *< 0.001, r_s _0.329), and corticosteroids (*p *< 0.001, r_s _0.245) but not with the use of epinephrine. This indicates that during the study period the use of dobutamine, norepinephrine, and corticosteroids significantly increased in the treatment of sepsis and purpura whereas the use of dopamine significantly decreased (Figure 5).

**Table 4:** Comparison of disease characteristics between children with sepsis and purpura before and after the national meningococcal C vaccination campaign (July 2002)

	Before meningococcal C vaccination^a^	After meningococcal C vaccination^a^
Total number of children (%)	248	39
	(86.4)	(13.6)
Case fatality (%)	45^b^	0^b^
	(18.1)	(0)
Age in years	3.2	2.5
	(0.1 to 17.9)	(0.3 to 13.1)
Number of children with DIC (%)	186^c^	20^c^
	(79.5)	(62.5)
Number of PICU-free days	24^c^	25^c^
	(0 to 28)	(0 to 27)
PRISM score	15	20
	(1 to 44)	(2 to 37)
Predicted death rate (%)^d^	5.6	8.1
	(0 to 100)	(0 to 100)
Base excess (mmol/L)	-7.7	-8
	(-28 to 4.4)	(-18 to -2)
Lactate (mmol/L)	4.1	4.0
Geometric mean, 95% CI	3.8 to 4.4	3.3 to 4.8
C-reactive protein (mg/L)	93	84
	(6 to 326)	(25 to 334)
Fibrinogen (g/L)	2.5	3.2
	(0.2 to 6.8)	(0.3 to 6.4)
Platelet count (×10^3^/μL)	110	135
	(13 to 475)	(25 to 227)
Leukocytes (×10^3^/μL)	8.9	12.1
Geometric mean, 95% CI	7.9 to 10.0	9.4 to 15.6
Glucose (mmol/L)	5.7^c^	7.2^c^
Geometric mean, 95% CI	5.3 to 6.2	6.2 to 8.2

^a^Results represent median (min-max) unless stated otherwise. ^b^*p *< 0.01. ^c^*p *< 0.05. ^d^Predicted death rate was based on the Rotterdam score. CI, confidence interval; DIC, disseminated intravascular coagulation; PICU, paediatric intensive care unit; PRISM, Paediatric Risk of Mortality.

## Discussion

In this monocenter cohort study of 287 children between the ages of 0 and 18 years with sepsis and purpura, we found that younger children had more severe disease and an increased risk of case fatality. The CFR of sepsis and purpura has improved in recent years despite comparable disease severity on admission. Male patients had higher PRISM scores and fewer PICU-free days. However, the CFR did not differ between males and females. Ethnicity did not influence disease severity and survival. The serogroups of *N. meningitidis *were not related to severity or survival.

Children with sepsis and purpura admitted to the PICU of the Erasmus MC-Sophia Children's Hospital account for approximately 25% of all paediatric sepsis cases in The Netherlands and therefore may provide a representative sample of cases in The Netherlands (National PICU registry, unpublished data). In addition, Rotterdam covers an area in The Netherlands (that is, the southwest of The Netherlands) in which meningococcal disease used to occur frequently.

In this large cohort of paediatric sepsis and purpura, low age was significantly associated with increased severity of disease, higher incidence of DIC, and increased CFR. Half of the children in our population were younger than 3 years of age. A comparison with the literature showed that incidence rates indeed decline after infancy and then increase again slightly during adolescence [1,9,24]. The increased CFR and the more severe disease in younger children may result from the still-developing immune, coagulation, and stress response systems in young children and therefore from the relative inability of young children to induce an effective immune response to a high load of micro-organisms such as *N. meningitidis *[13,19].

The CFR due to sepsis and purpura was 15.7% over the past two decades. This is in accordance with other large studies reporting CFRs of 10.4% to 20% [7,24-26]. It must be noted that Jensen and colleagues [7] and Sharip and colleagues [24] studied meningococcal disease, not specifically paediatric sepsis and purpura.

*N. meningitidis *was the causative organism of sepsis and purpura in the vast majority of cases. Martin and colleagues [27] also found that Gram-negative bacteria were the predominant causative organisms of sepsis in the US between 1979 and 1987. In our study, the incidence of disease due to serogroup B was much higher than that due to serogroup C. Serogroup B *N. meningitidis *was seen more often in younger children compared with serogroup C. No differences with respect to severity of illness scores and CFR were observed between serogroups B and C. Erickson and De Wals [28] suggested a more severe course of serogroup C infections, indicated by increased mortality due to serogroup C (14%) compared with serogroup B (7%). Spanjaard and colleagues [29] found a CFR in meningococcal sepsis caused by serogroup B of 8.1% compared with 7.1% in serogroup C. However, Erickson and De Wals [28] studied both meningitis and sepsis in all culture-proven cases of *N. meningitidis*, and Spanjaard and colleagues [29] studied all culture-proven cases including adults in The Netherlands, whereas we studied paediatric cases of sepsis and purpura.

Since the implementation of the meningococcal C vaccination in July 2002, there has not been a fatal case of sepsis and purpura in our PICU. Because severity of disease before and after the implementation did not differ between the two groups, the increased survival may have resulted from improved treatment strategies [8]. International treatment guidelines were implemented at that time, health care workers received additional training, and public awareness increased, resulting in a decreased patient delay. Furthermore, we observed a change in the choice of inotropic agents used since 2002. It must be noted that the number of children included since 2002 is low. However, these observations do warrant further research in a prospective study.

Gender was not associated with CFR from sepsis and purpura although males did have significantly more severe disease, based on the PRISM score and fewer PICU-free days, compared with females. Bindl and colleagues [30] found a male-to-female ratio of 1.7 in sepsis patients ages 1 week to 8 years with severe sepsis and septic shock, whereas we observed a male-to-female ratio of 1.2. However, in those cases caused by *N. meningitidis*, which is the major causative organism in our study, males and females were represented equally among non-survivors. Watson and colleagues [26] and Martin and colleagues [27] also found a predisposition for male gender in sepsis, but they did not specify the male-to-female ratio in sepsis caused by *N. meningitidis*.

Due to the small number of children in the different ethnic groups, we may not have been able to detect differences between the different ethnic groups with respect to severity or case fatality of sepsis and purpura. In addition, during the 18-year study period, the dynamics of the Dutch population (especially in Rotterdam) underwent changes, which may not be reflected in this study. Rosenstein and colleagues [1] proposed a predisposition for sepsis in children of African descent. Sharip and colleagues [24] found an age-adjusted increased risk of case fatality in individuals of African descent compared with Caucasians and other ethnic groups.

A possible limitation of our study may be that the serotypes of *N. meningitidis *were not determined in all children with meningococcal sepsis. Due to the rapidly progressive nature of this disease, it is possible that we did not include a number of the most severe cases because of case fatality before admission or referral to the Erasmus MC-Sophia Children's Hospital. On the other hand, the fact that only children with sepsis and purpura admitted to the PICU were included may have resulted in a skewed representation of all children with sepsis and purpura (that is, children with relatively mild disease admitted to a general ward).

## Conclusion

The CFR in this study was 15.7%. Age was the most important predictor of severity and case fatality of sepsis and purpura. Male gender was associated with higher PRISM scores and fewer PICU-free days, but no differences in CFR were seen. *N. meningitidis *was the causative organism in the vast majority of cases. No differences between *N. meningitidis *serogroups B and C with respect to disease severity scores and case fatality were observed. Ethnicity was not associated with the course of sepsis and purpura.

In future studies investigating effects on severity and survival of sepsis and purpura, age and gender should be taken into account. The possible effect of the change in choice of inotropic agents warrants further investigation. Also, other possible differences between male and female patients with sepsis should be investigated. With the changing demography in The Netherlands (especially in the Rotterdam area), differences between ethnic groups require further examination.

## Key messages

• Mortality of children with sepsis and purpura improved substantially from 1988 to 2006. A possible explanation is an improvement in supportive treatment.

• Younger children (below 3 years of age) have a more severe disease state and a higher risk of case fatality than older children.

• Male patients have a more severe disease according to disease severity scoring systems, but this has not led to increased mortality in this group of 287 children.

• The major causative organism of sepsis and purpura in children is *Neisseria meningitidis*. Since the introduction of the vaccination in 2002, *N. meningitidis *serogroup C has completely vanished as a causative organism.

• Serogroup of *N. meningitidis *and ethnicity were not associated with the course of disease in children with sepsis and purpura.

## Abbreviations

CFR = case fatality rate; CI = confidence interval; CRP = C-reactive protein; DIC = disseminated intravascular coagulation; PDR = predicted death rate; PICU = paediatric intensive care unit; PRISM = Paediatric Risk of Mortality; r_s _= Spearman correlation coefficient.

## Competing interests

The authors declare that they have no competing interests.

## Authors' contributions

MM participated in creating the database, performed the statistical analysis, and wrote the manuscript. CMPB assisted in creating the database, interpretation of the results, and the writing of the manuscript. ME assisted in creating the database, the statistical analysis, interpretation of the results, and the writing of the manuscript. LS was responsible for *N. meningitidis *serogrouping and serotyping and critically read the manuscript. KFMJ critically read the manuscript and assisted in interpretation of the results. RdG assisted in the writing of the manuscript and was responsible for the studies in which the patients were included. JAH was also responsible for the studies in which the patients were included, initiated this study, and assisted in the statistical analysis, interpretation of the results, and the writing of the manuscript. All authors read and approved the final manuscript.

## References

[B1] Rosenstein NE, Perkins BA, Stephens DS, Popovic T, Hughes JM (2001). Meningococcal disease. N Engl J Med.

[B2] Cohen J (2002). The immunopathogenesis of sepsis. Nature.

[B3] Hazelzet JA (2005). Diagnosing meningococcemia as a cause of sepsis. Pediatr Crit Care Med.

[B4] Vermont CL, de Groot R, Hazelzet JA (2002). Bench-to-bedside review: genetic influences on meningococcal disease. Crit Care.

[B5] Guarner J, Greer PW, Whitney A, Shieh WJ, Fischer M, White EH, Carlone GM, Stephens DS, Popovic T, Zaki SR (2004). Pathogenesis and diagnosis of human meningococcal disease using immunohistochemical and PCR assays. Am J Clin Pathol.

[B6] Emonts M, Hazelzet JA, de Groot R, Hermans PW (2003). Host genetic determinants of *Neisseria meningitidis *infections. Lancet Infect Dis.

[B7] Jensen ES, Schonheyder HC, Lind I, Berthelsen L, Norgard B, Sorensen HT (2003). *Neisseria meningitidis *phenotypic markers and septicaemia, disease progress and case-fatality rate of meningococcal disease: a 20-year population-based historical follow-up study in a Danish county. J Med Microbiol.

[B8] Booy R, Habibi P, Nadel S, de Munter C, Britto J, Morrison A, Levin M, Meningococcal Research Group (2001). Reduction in case fatality rate from meningococcal disease associated with improved healthcare delivery. Arch Dis Child.

[B9] (2002). Netherlands Reference Lab for Bacterial Meningitis. Bacterial Meningitis in The Netherlands 2001.

[B10] (2003). Netherlands Reference Lab for Bacterial Meningitis. Bacterial Meningitis in The Netherlands 2002.

[B11] Vermont CL, den Brinker M, Kâkeci N, de Kleijn ED, de Rijke YB, Joosten KF, de Groot R, Hazelzet JA (2005). Serum lipids and disease severity in children with severe meningococcal sepsis. Crit Care Med.

[B12] den Brinker M, Joosten KF, Liem O, de Jong FH, Hop WC, Hazelzet JA, van Dijk M, Hokken-Koelega AC (2005). Adrenal insufficiency in meningococcal sepsis: bioavailable cortisol levels and impact of interleukin-6 levels and intubation with etomidate on adrenal function and mortality. J Clin Endocrinol Metab.

[B13] de Groof F, Joosten KF, Janssen JA, de Kleijn ED, Hazelzet JA, Hop WC, Uitterlinden P, van Doorn J, Hokken-Koelega AC (2002). Acute stress response in children with meningococcal sepsis: important differences in the growth hormone/insulin-like growth factor I axis between nonsurvivors and survivors. J Clin Endocrinol Metab.

[B14] de Kleijn ED, de Groot R, Hack CE, Mulder PG, Engl W, Moritz B, Joosten KF, Hazelzet JA (2003). Activation of protein C following infusion of protein C concentrate in children with severe meningococcal sepsis and purpura fulminans: a randomized, double-blinded, placebo-controlled, dose-finding study. Crit Care Med.

[B15] Derkx B, Wittes J, McCloskey R (1999). Randomized, placebo-controlled trial of HA-1A, a human monoclonal antibody to endotoxin, in children with meningococcal septic shock. European Pediatric Meningococcal Septic Shock Trial Study Group. Clin Infect Dis.

[B16] Van der Kaay DC, De Kleijn ED, De Rijke YB, Hop WC, De Groot R, Hazelzet JA (2002). Procalcitonin as a prognostic marker in meningococcal disease. Intensive Care Med.

[B17] Goldstein B, Giroir B, Randolph A (2005). International pediatric sepsis consensus conference: definitions for sepsis and organ dysfunction in pediatrics. Pediatr Crit Care Med.

[B18] Zuppa AF, Nadkarni V, Davis L, Adamson PC, Helfaer MA, Elliott MR, Abrams J, Durbin D (2004). The effect of a thyroid hormone infusion on vasopressor support in critically ill children with cessation of neurologic function. Crit Care Med.

[B19] Kornelisse RF, Hazelzet JA, Hop WC, Spanjaard L, Suur MH, van der Voort E, de Groot R (1997). Meningococcal septic shock in children: clinical and laboratory features, outcome, and development of a prognostic score. Clin Infect Dis.

[B20] Pollack MM, Ruttimann UE, Getson PR (1988). Pediatric risk of mortality (PRISM) score. Crit Care Med.

[B21] Taylor FB, Toh CH, Hoots WK, Wada H, Levi M (2001). Towards definition, clinical and laboratory criteria, and a scoring system for disseminated intravascular coagulation. Thromb Haemost.

[B22] Bouwhuis CB, Moll HA (2003). Determination of ethnicity in children in The Netherlands: two methods compared. Eur J Epidemiol.

[B23] van der Ende A, Schuurman IG, Hopman CT, Fijen CA, Dankert J (1995). Comparison of commercial diagnostic tests for identification of serogroup antigens of *Neisseria meningitidis*. J Clin Microbiol.

[B24] Sharip A, Sorvillo F, Redelings MD, Mascola L, Wise M, Nguyen DM (2006). Population-based analysis of meningococcal disease mortality in the United States: 1990–2002. Pediatr Infect Dis J.

[B25] van Deuren M, Brandtzaeg P, van der Meer JW (2000). Update on meningococcal disease with emphasis on pathogenesis and clinical management. Clin Microbiol Rev.

[B26] Watson RS, Carcillo JA, Linde-Zwirble WT, Clermont G, Lidicker J, Angus DC (2003). The epidemiology of severe sepsis in children in the United States. Am J Respir Crit Care Med.

[B27] Martin GS, Mannino DM, Eaton S, Moss M (2003). The epidemiology of sepsis in the United States from 1979 through 2000. N Engl J Med.

[B28] Erickson L, De Wals P (1998). Complications and sequelae of meningococcal disease in Quebec, Canada, 1990–1994. Clin Infect Dis.

[B29] Spanjaard L, Bol P, de Marie S, Zanen HC (1987). Association of meningococcal serogroups with the course of disease in the Netherlands, 1959–83. Bull World Health Organ.

[B30] Bindl L, Buderus S, Dahlem P, Demirakca S, Goldner M, Huth R, Kohl M, Krause M, Kühl P, Lasch P, ESPNIC ARDS Database Group (2003). Gender-based differences in children with sepsis and ARDS: the ESPNIC ARDS Database Group. Intensive Care Med.

